# Game-Changing Innovations: How Culture Can Change the Parameters of Its Own Evolution and Induce Abrupt Cultural Shifts

**DOI:** 10.1371/journal.pcbi.1005302

**Published:** 2016-12-30

**Authors:** Oren Kolodny, Nicole Creanza, Marcus W. Feldman

**Affiliations:** 1 Department of Biology, Stanford University, Stanford, California, United States of America; 2 Department of Biological Sciences, Vanderbilt University, Nashville, Tennessee, United States of America; The University of Tennessee, UNITED STATES

## Abstract

One of the most puzzling features of the prehistoric record of hominid stone tools is its apparent punctuation: it consists of abrupt bursts of dramatic change that separate long periods of largely unchanging technology. Within each such period, small punctuated cultural modifications take place. Punctuation on multiple timescales and magnitudes is also found in cultural trajectories from historical times. To explain these sharp cultural bursts, researchers invoke such external factors as sudden environmental change, rapid cognitive or morphological change in the hominids that created the tools, or replacement of one species or population by another. Here we propose a dynamic model of cultural evolution that accommodates empirical observations: without invoking external factors, it gives rise to a pattern of rare, dramatic cultural bursts, interspersed by more frequent, smaller, punctuated cultural modifications. Our model includes interdependent innovation processes that occur at different rates. It also incorporates a realistic aspect of cultural evolution: cultural innovations, such as those that increase food availability or that affect cultural transmission, can change the parameters that affect cultural evolution, thereby altering the population’s cultural dynamics and steady state. This steady state can be regarded as a *cultural carrying capacity*. These parameter-changing cultural innovations occur very rarely, but whenever one occurs, it triggers a dramatic shift towards a new cultural steady state. The smaller and more frequent punctuated cultural changes, on the other hand, are brought about by innovations that spur the invention of further, related, technology, and which occur regardless of whether the population is near its cultural steady state. Our model suggests that common interpretations of cultural shifts as evidence of biological change, for example the appearance of behaviorally modern humans, may be unwarranted.

## Author Summary

The ability to accumulate culture is unique to hominids. Yet, understanding of the processes that drive cultural evolution is limited. We develop a computational model that incorporates characteristics of human innovation and offers an explanation for one of the puzzling observations in the archaeological record of stone tools: its extreme punctuation. The record shows long periods of little technological change, interspersed with abrupt increases in cultural complexity. Within these periods of relative stasis smaller punctuations occur, with sudden gain or loss of suites of tools. We suggest that the two types of punctuation may result from two processes: small punctuations are driven by innovations that trigger invention of related tools, and large punctuations reflect changes in the steady state of the size of the population’s tool repertoire. In our model, these changes of steady state occur when cultural processes can change the parameters of their own evolution. This occurs, for example, through innovations that change the availability of food and lead to an increased population size or innovations that increase the effectiveness of cultural transmission. Our results suggest that the common attribution of sudden cultural shifts to external processes such as cognitive or environmental change may be unwarranted.

## Introduction

The archaeological record indicates that cultural traits can accumulate exponentially over time [1–3] and dramatic cultural losses can also occur [4–7]. However, depending on the timescale studied and the time-resolution in which it is analyzed, changes in tool repertoire may appear punctuated and stepwise; for example, in the prehistoric archaeological record, long periods of little change are separated by “cultural explosions,” brief periods of rapid cultural accumulation [3,4,8–15]. Reasons for the sudden changes in cultural repertoires continue to be debated, but this pattern of change has usually been attributed to external events, such as environmental changes or the evolution of new cognitive capacities [16,17]. Importantly, upon close scrutiny, the punctuation in some cultural records exhibits an additional characteristic: within the long periods of seemingly little broad-scale cultural change, there are cultural shifts of a smaller scale. In the prehistoric record of stone tools, for example, there are long periods of little change, interspersed with sudden dramatic cultural shifts, such as the Neolithic revolution or the transition between the Middle and Upper Paleolithic [11,18]. However, within each of these periods of relative stasis, smaller punctuations have been detected between relatively short periods of stasis [19–21]. Existing mathematical models do not account for these patterns of punctuation.

Explanations of punctuated patterns of cultural accumulation that invoke external changes entail an implicit assumption that the size of the cultural repertoire is generally at some steady state, and that a burst of change in this repertoire is due to an external force that alters this steady state, such as environmental [17,22] or cognitive [16,23–25] change. In contrast to these bursts in cultural evolution, punctuated equilibria in *biological* evolution [26–31] have been hypothesized to be intrinsic to the evolutionary process itself, not necessarily because of external mechanisms [32].

In this vein, our recent theoretical model of innovation and cultural accumulation [33] demonstrated that periods between bursts of change in the cultural repertoire could reflect waiting times between ground-breaking innovations that facilitate the invention and accumulation of other, related, innovations. In other words, punctuation in the cultural record could be an emergent property of cultural accumulation itself. Notably, in this recent model and in other models of cultural evolution, the time trajectory of the cultural repertoire’s size approaches a steady state that is dependent on system parameters, such as population size, rate of invention, and rate of loss of innovations [33–38].

In the current study we combine within the same model two paradigms of what may drive punctuated cultural change: (i) shifts in the population’s cultural steady state, in terms of its cultural carrying capacity, which trigger rapid cultural accumulation, and (ii) the occurrence of groundbreaking innovations that trigger the invention of related technology. This gives rise to a framework in which cultural bursts can occur on two timescales and are characterized by two scales of magnitude. Change in the population’s cultural carrying capacity, the size of its tool repertoire at equilibrium, occurs very rarely, following a cultural innovation that directly alters the parameters of cultural evolution. This can occur, for example, if a tool increases food availability and thus the *biological carrying capacity* of the habitat (the number of hominid individuals that the available resources can support), leading to an increase in the population size, or if a cultural innovation increases the efficacy of cultural transmission, reducing the rate of cultural loss. Change of the cultural steady state, the size of the cultural repertoire at equilibrium, typically induces rapid cultural accumulation of a large magnitude (henceforth *major shifts*). Innovations that trigger invention of related technology occur in our framework more frequently, and typically involve relatively small increases in the cultural repertoire (*minor shifts*). Importantly, both types of punctuation are intrinsic to the process of cultural evolution itself, and do not require invocation of external factors.

### The model

We investigate not only the effects of innovation processes on the cultural repertoire, but also ways in which these processes can fundamentally change the dynamics of cultural accumulation. Here we provide a description of the dynamic model and, under simplifying assumptions, some analytical derivations of the *expected* number of tools arising from the three interacting processes (see also SI section 2 of [33]). We implement this model as an agent-based stochastic simulation.

In the model of Kolodny *et al*. [33], which we reframe and expand in the current study, tool innovation consists of three interacting stochastic processes. The first process consists of invention of large-scale innovations. These are also called *lucky leaps*, and are stochastically added to a population of size *N* with probability *P*_lucky_ per individual per time step with an expected rate of change of $\frac{\Delta n_{lucky}}{\Delta t}=P_{lucky}\cdot N$. In a population that starts out with a cultural repertoire of size zero, the expected number of these lucky leaps at time *t* can thus be written as

$$n_{lucky}=P_{lucky}∙N∙t.$$ (1)

These large-scale innovations facilitate two other innovation processes, allowing the accumulation of two additional types of tools: *toolkit innovations*, and *innovative combinations*. First, each lucky leap can be associated with *L* toolkit innovations, which are tools that are made useful by the existence of the lucky leap, where *L* is sampled from a uniform distribution *U*(1,*L*_*max*_). The expected rate of change of toolkit innovations is $\frac{\Delta n_{toolkit}}{\Delta t}=P_{lucky}\cdot N\cdot \left\langle L\right\rangle$, where ⟨*L*⟩ denotes the mean value of toolkit sizes, i.e. $\frac{1+L_{max}}{2}$. With both lucky leaps and toolkit innovations, the expected potential size of this tool repertoire at time *t* is given as:

$$n_{lucky}+n_{toolkit}=P_{lucky}∙N∙t+P_{lucky}∙\langle L\rangle ∙N∙t.$$ (2)

In the stochastic simulations, this potential number of toolkit innovations often accumulates over multiple time steps. Each individual has a probability *P*_*toolkit*_ of producing a toolkit innovation per time step; if *P*_*toolkit*_ or *N* is large, the full potential size of a lucky leap’s toolkit is quickly reached once there is a lucky leap.

Second, a lucky leap can be combined with another tool to produce an innovative combination, which is useful to the population with probability *P*_*combUseful*_. With this type of innovation included, and considering for simplicity only combinations of lucky leap tools, the expected rate of change of the number of combination tools per time step is $\frac{\Delta n_{comb}}{\Delta t}=P_{lucky}\cdot N\cdot n_{lucky}\cdot P_{combUseful}$.

Summing the expected number of lucky leaps, toolkit innovations, and combinations at time *t* gives:

$$n_{lucky}+n_{toolkit}+n_{comb}=P_{lucky}∙N∙t+P_{lucky}∙L∙N∙t+½{\left(P_{lucky}∙N\right)}^{2}∙P_{combUseful}∙t^{2}.$$ (3)

As mentioned above, the analytical derivations presented in Eqs 1–3 allow us to calculate the *potential* number of tools expected at a given time step under the simplifying assumption that all toolkit and combination innovations are immediately tested when a new lucky leap is invented. In the stochastic simulations, each individual has a probability *P*_*combine*_ in each time step of combining two tools to check whether this gives rise to a useful tool.

At each time step, tools can also be randomly lost, which occurs with probability *P*_*SpontLoss*_*/N*, since we expect the rate of cultural loss to decrease as population size increases. Toolkit innovations and combination tools are lost in our model if the lucky leap tool with which they are associated is lost. With probability $\frac{n_{lucky}}{n_{lucky}+n_{toolkit}+n_{comb}}$, the tool that is lost is a lucky leap, and thus its associated toolkit and combinations are lost with it, so when a lucky leap is lost, the total number of tools lost is *L*_*t*_ + *C*_*t*_ + 1, where *L*_*t*_ and *C*_*t*_ are, respectively, the mean number of toolkit innovations and combination innovations associated with a lucky leap innovation at the time of its loss, *t*, and the 1 accounts for the lucky leap itself. With probability $1-\frac{n_{lucky}}{n_{lucky}+n_{toolkit}+n_{comb}}$, the tool lost is a toolkit or combination innovation, and the number of tools lost is 1. Thus, at each time step, there is an expected loss term that is subtracted from the number of tools gained at that step:

$$-\frac{P_{SpontLoss}}{N}\cdot n_{total}\cdot \left(\frac{n_{lucky}}{n_{total}}\cdot \left(L_{t}+C_{t}+1\right)+\left(1-\frac{n_{lucky}}{n_{total}}\right)\right),$$ (4a)

where *n*_*total*_ = *n*_*lucky*_ + *n*_*toolkit*_ + *n*_*comb*_. Making this substitution gives the loss term:

$$-P_{SpontLoss}/N\cdot \left(n_{lucky}+n_{toolkit}+n_{comb}\right)-P_{SpontLoss}/N\cdot n_{lucky}\cdot L_{t}-P_{SpontLoss}/N\cdot n_{lucky}\cdot C_{t}$$ (4b)

Simply by accounting for multiple interacting innovation processes, this model produces punctuated bursts of cultural innovations after periods of stasis, since the stochastic addition of a new lucky leap can facilitate the addition of numerous combinations and toolkit innovations on a relatively short timescale [33]. When stochastic innovation and spontaneous loss both occur in the model, the number of tools in the population’s repertoire eventually approaches a steady state. To describe the number of tools at steady state, Kolodny *et al*. derive the following equations for each type of tool under some simplifying assumptions. These describe the asymptote of the curve, and represent the expected number of tools from each type in a scenario that considers spontaneous loss (see also SI of [33]):

$$n_{lucky}^{*}=\frac{N^{2}\cdot P_{lucky}}{P_{SpontLoss}}$$ (5)

$$n_{toolkit}^{*}=\frac{N^{2}\cdot P_{lucky}\cdot <L>}{2\cdot P_{SpontLoss}}$$ (6)

$$n_{comb}^{*}=\frac{N^{4}\cdot P_{lucky}{}^{2}\cdot P_{CombUseful}}{2\cdot P_{SpontLoss}{}^{2}}$$ (7)

In the model described so far, periods of stasis in the cultural record represent the waiting times between large-scale innovations, which can then facilitate the accumulation of many other innovations through other pathways. In other words, some human innovations require large leaps of insight, but other innovations can be created by drawing parallels with existing technologies or by combining existing technologies to make a new tool. These different processes of innovation occur at different rates, and the relationships between them and their rates determine whether the accumulation of tools occurs in a punctuated pattern. However, with given rates of innovation and loss, a population’s number of tools eventually reaches a steady state in which there is a stochastic balance between the loss and accumulation of tools in the population.

The idea of this cultural steady state suggests an alternative explanation for the dramatic bursts observed in the cultural record: periods of stasis could be stretches of time in which the population is at steady state in its cultural evolution, and extensive cultural change could occur following a change in one or more of the parameters that determine the cultural carrying capacity (the size of the tool repertoire at a steady state). In other words, if processes change one or more of the parameters in Eqs (5)–(7), we would expect a major punctuated shift to a new steady state. Changes in these parameters could be a result of changes in extrinsic factors, as has been suggested in the literature to explain punctuated cultural shifts. For example, the increase in cognitive capacity that Klein [16] suggested as an explanation for the cultural burst ~50kya could be represented in our model’s terms as an increase in *P*_*lucky*_. We suggest a parsimonious and realistic alternative to the explanation of cultural bursts as responses to cognitive or environmental changes: a punctuated shift in the steady state could also result from factors intrinsic to cultural evolution itself, such as the spread of a game-changing innovation that alters the parameters of cultural evolution. Here, we explore two particular processes that could give rise to such parameter changes.

### Increase in population size

First, we account for the possibility that a rare cultural trait might foster an increase in the biological carrying capacity of the habitat and hence of the population size. For example, the invention of certain agricultural techniques might lead to increased crop yields and thus produce an increase in population size [39]. According to Eqs (5)–(7), such an increase is expected to result in a larger cultural repertoire at steady state. Population size and cultural repertoire have been linked in both empirical (e.g. [40–47] but see [48,49]) and theoretical studies (e.g. [5,33,34,50–52], but see the debate in [53,54]).

At each time step in our model, a lucky leap occurs with probability *P*_*lucky*_ per individual. Each such lucky leap is assumed to have a probability *P*_*ToolChangesCarryingCapacity*_*/N* of turning out to be an innovation that alters the biological carrying capacity of the system. If this occurs at time *t*, we assume that the increase in the population size is carried out by multiplying the current population size, *N*, by a number *T* sampled from a uniform distribution:

$$N_{t}{}_{+1}=N_{t}∙T$$ (8)

In the results presented here, we chose a range for *T* of *U*(1.2,1.6) for visual clarity of the figures: with the parameters of cultural evolution used in our simulations, population size changes by factors of 1.2 to 1.6 lead to visually clear changes in the cultural steady state, but do so without suddenly changing the steady state’s order of magnitude. Choosing a different range for *T* does not qualitatively change the results. After this population size increase, with no change in other parameters of the model, the population can accumulate more tools, and the number of tools in the cultural repertoire increases. Analytically, the steady state is linearly dependent on the rate of lucky leap innovation (*P*_*lucky*_), quadratically dependent on the population size (~*N*^2^), and inversely proportional to rate of loss (~1/*P*_*SpontLoss*_); see Eq 6. These relationships are sensitive to the details of the model; for example, if innovative combinations are included, *P*_*lucky*_ varies as ~*N*^4^ (Eq 7).

If the lucky leap innovation associated with the increased biological carrying capacity is lost in the population, the population size can revert to its previous level, and the tool repertoire will subsequently drop as well. In realistic terms, this would be manifested as a change back to the population’s original subsistence strategy.

As noted above, the probability that a lucky leap innovation leads to a change in biological carrying capacity is dependent on *P*_*ToolChangesCarryingCapacity*_, but is also assumed to be inversely proportional to the population size at the time of its invention. The reasoning behind this choice is that as the population is larger, it is more likely to already be making efficient use of a larger proportion of the habitat’s resources, making it harder to invent a means of increasing the resources available that would translate to an increased biological carrying capacity. In reality, the relationship between population size and the likelihood of increasing biological carrying capacity may be nonlinear and possibly involve additional factors; we choose this inverse relationship for simplicity (but see discussion for an alternative). Because the rate of lucky leap innovations is dependent linearly on population size, there is a constant expected rate of occurrence of innovations that increase biological carrying capacity, independent of population size.

### Decrease in the rate at which culture is lost

Second, we modified our model [33] to account for the possibility that a rare cultural trait might cause a decrease in the rate at which culture is lost. For example, the invention of a writing system [55] or other ways of preserving cultural information might lead to a lower probability that a trait is forgotten. A decrease in the rate at which innovations are lost effectively increases trait retention and thus results in an increased cultural repertoire size without altering the population size. With probability *P*_*LossRateReduction*_, a lucky leap innovation reduces the loss rate. When this occurs, the loss rate, *P*_*SpontLoss*_, at time *t*+1 is that at time *t* multiplied by a number *S* sampled from a uniform distribution:

$$P_{SpontLoss}{}_{\left(t+1\right)}=P_{SpontLoss}{}_{\left(t\right)}∙S.$$ (9)

As with the factor of population size increase, *T*, a range for *S* of *U*(0.5,0.9) was chosen for visual clarity of the presented results. Choice of a different range does not qualitatively change the results.

With probability *P*_*reverse*_ per time step, an innovation that decreased *P*_*SpontLoss*_ is itself lost, and *P*_*SpontLoss*_ reverts to its previous value and tools are lost with higher probability in the population.

## Results

Here, we analyze a model that encompasses two realistic ways in which innovations can affect the trajectory of cultural evolution: one in which a large-scale innovation, for example a more effective subsistence strategy, can alter the population size and thus the cultural steady state of the population, and one in which a large-scale innovation, such as writing or other mechanisms of preserving cultural information, can affect the rate of cultural loss. With all external factors remaining constant in our previous model [33], a population’s cultural gains and losses reach an eventual cultural steady state, such that the rates of cultural innovation and loss balance one another and the population maintains a relatively stable number of tools. By altering the parameters that affect cultural gains and losses, game-changing innovations in human culture can perturb the stochastic steady state, resulting in large-scale cultural bursts. In the simulations presented here, we restrict the possible innovation processes to lucky leaps and toolkit innovations for simplicity. The results of simulations that include innovative combinations are qualitatively similar, but the dynamics are more difficult to visualize because tools accumulate much more rapidly.

**Fig 1:**
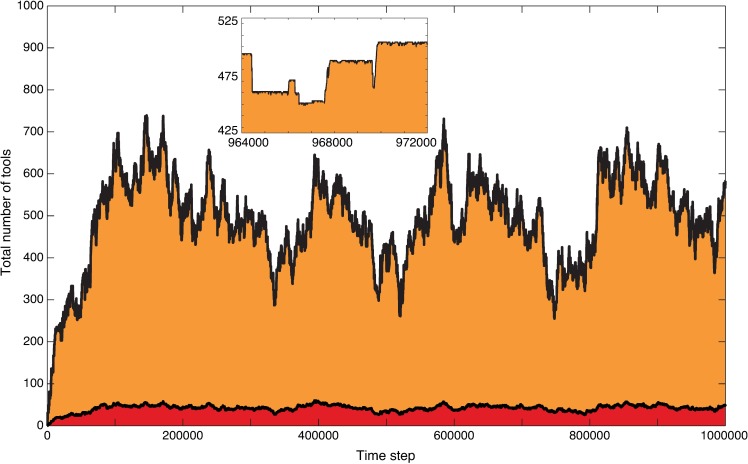
The time trajectory of the cultural repertoire when the parameters of cultural evolution do not change, leading to a constant cultural carrying capacity. When no innovations that change the parameters determining cultural evolution are allowed, the cultural repertoire (total number of tools) initially grows and stabilizes in a dynamic equilibrium around the cultural carrying capacity. The fluctuations in the repertoire size are driven by invention and loss of single toolkit tools and of lucky leap tools with their associated toolkits. The latter lead to minor punctuated changes in the size of the cultural repertoire, interspersed with periods of near-stasis. Red indicates lucky leap innovations and orange indicates toolkit innovations. The inset is an enlarged version of the repertoire sizes between time steps 964000 and 972000, showing these minor shifts. Parameters: *N* = 20, *P*_*lucky*_ = 0.0008, *P*_*toolkit*_ = 1, *P*_*combine*_ = 0, *P*_*spontLoss*_ = 0.0008, *P*_*ToolChangesCarryingCapacity*_ = 0, *L*_*max*_ = 21.

**Fig 1** is an example of a time trajectory of a cultural repertoire under the assumption that innovations *do not* change the parameters of cultural evolution. The punctuation in this trajectory (see inset) is thus driven exclusively by lucky leap innovations, which trigger further invention of related toolkit innovations, corresponding to the minor cultural punctuations discussed above. The repertoire initially grows in size, and stabilizes on a stochastic equilibrium near a repertoire size of 480 tools around which large fluctuation can be seen, driven by losses and inventions of tools. A decrease of a single tool in the cultural repertoire is typically a result of stochastic loss of a toolkit innovation, while instantaneous loss of multiple tools is the result of stochastic loss of a lucky leap innovation, which in our model results in the loss of its associated toolkit innovations as well.

**Fig 2:**
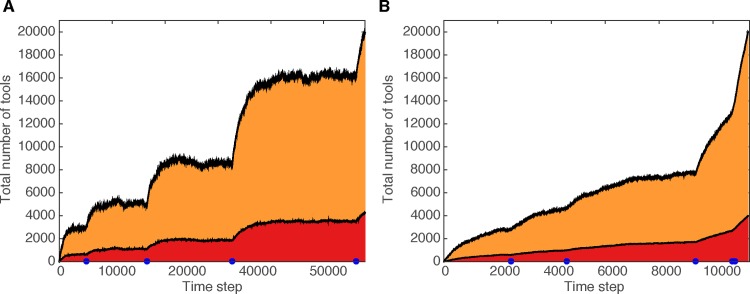
Cultural accumulation when innovations may alter subsistence strategy, increasing biological carrying capacity and leading to an increase in population size. Red indicates lucky leap innovations and orange indicates toolkit innovations. Blue dots indicate the occurrence of rare innovations that alter the biological carrying capacity. The cultural trajectories in panels **A** and **B** share the same underlying parameters, but stochastic differences between simulations led panel **A** to have longer time intervals between the occurrence of innovations that altered the carrying capacity, resulting in punctuated bursts of innovations after periods of stasis. In contrast, panel **B,** by chance, has less time between innovations that alter biological carrying capacity, so the population does not approach the cultural steady state in these time intervals. For clarity of visualization, both cultural trajectories halt when the population reaches 20,000 tools in its cultural repertoire. Parameters: *N* (initial) = 80, *P*_*lucky*_ = 0.08, *P*_*toolkit*_ = 0.2, *P*_*combine*_ = 0, *P*_*spontLoss*_ = 0.08, *P*_*ToolChangesCarryingCapacity*_ = 0.002, *L*_*max*_ = 11.

**Fig 2** shows the effects of an innovation that alters the stochastic steady state of the cultural repertoire. At several time steps (indicated by blue dots on the *x*-axis), an innovation occurs that changes the biological carrying capacity and thus increases the population size. This is implemented by multiplying the population size, *N*, by a number drawn from a uniform distribution between 1.2 and 1.6. At each time step, there is a small probability, *P*_*ToolChangesCarryingCapacity*_*/N*, where *P*_*ToolChangesCarryingCapacity*_ = 0.002, that a lucky leap innovation alters the biological carrying capacity in this way. In some simulations, the number of tools in the population plateaus at the cultural steady state between each major punctuation event, and the pattern of cultural accumulation is punctuated and stepwise (**Fig 2A**). However, if by chance these changes in biological carrying capacity occur more frequently, the population does not have the opportunity to approach the steady states, and the increase in tool repertoire is more gradual and less punctuated (**Fig 2B**). Thus, even for the same underlying probability of this type of culturally induced change in the biological carrying capacity, a range of qualitative results is possible, from continuous to very punctuated changes.

Although the two cultural trajectories in **Fig 2** were simulated with the same set of parameters, a similar pattern can be produced by altering the probability that an innovation changes the biological carrying capacity. For example, for very low values of *P*_*ToolChangesCarryingCapacity*_, the biological carrying capacity changes rarely enough that the cultural repertoire nearly always reaches a steady state between these changes, whereas for higher values, these changes occur often enough that plateaus are very rare. The rates at which tools of different types are invented, as well as the rate of tool loss, affect the overall rate of tool accumulation. Thus, whether the cultural repertoire reaches a steady state between changes in the biological carrying capacity depends on the relations between all of the model parameters, and not only on *P*_*ToolChangesCarryingCapacity*_.

In **Fig 2**, we chose a single intermediate value of *P*_*ToolChangesCarryingCapacity*_ for both panels to demonstrate that the inherent stochasticity in the system can produce a spectrum of different qualitative patterns with the same underlying parameters. In the simulations presented in **Fig 2**, we did not allow the possibility of loss of an innovation that had led to an increase in biological carrying capacity, which is discussed in greater detail below.

**Fig 3:**
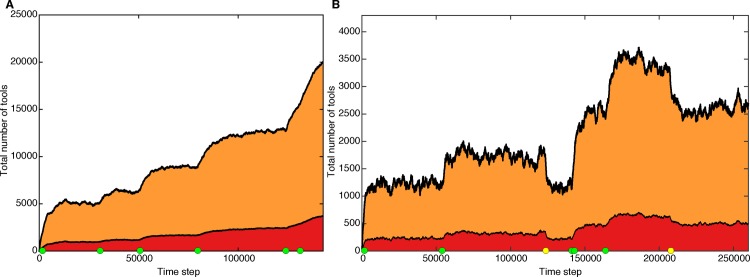
Cultural accumulation when innovations can increase and decrease the rate of culture loss. Red indicates lucky leap innovations and orange indicates toolkit innovations. A green dot indicates the occurrence of a rare innovation that decreases the rate at which culture is lost. The cultural trajectories in panels **A** and **B** differ in that panel **B** allows those loss-reducing innovations to be themselves lost, thus effectively increasing the rate of cultural loss. Losses of these innovations are indicated by yellow dots. Note that the *y*-axes on panels **A** and **B** differ greatly. Panel **A** parameters: *N* = 80, *P*_*lucky*_ = 0.04, *P*_*toolkit*_ = 0.2, *P*_*combination*_ = 0, *P*_*spontLoss*_ (initial) = 0.04, *P*_*LossRateReduction*_ = 0.00002, *P*_*reverse*_ = 0, *L*_*max*_ = 11. Panel **B** parameters: *N* = 40, *P*_*lucky*_ = 0.04, *P*_*toolkit*_ = 0.2, *P*_*combine*_ = 0, *P*_*spontLoss*_ (initial) = 0.04, *P*_*LossRateReduction*_ = 0.000007, *P*_*reverse*_ = 0.000004, *L*_*max*_ = 11.

**Fig 3** shows the effects of innovations that alter the rate of cultural loss. In this scenario, there is a small probability that a lucky leap innovation reduces the rate of stochastic cultural loss; for example, a writing technology or techniques for accurate transmission of oral tradition. With probability *P*_*LossRateReduction*_, the stochastic rate of cultural loss, *P*_*SpontLoss*_, is multiplied by a number drawn from a uniform distribution between 0.5 and 0.9, as shown by the green dots in **Fig 3**. This alteration in the loss rate changes the stochastic cultural steady state of the population, and the cultural repertoire increases. Because of the stochastic nature of these innovations, multiple changes can occur within a short time period, resulting in an even more dramatic burst, such as in **Fig 3B** near time step 142,000. With probability *P*_*reverse*_ per time step this loss-reducing technology is itself lost, as shown by the yellow dots in **Fig 3B**, leading to a corresponding decrease in the number of tools accumulated.

## Discussion

The trajectory of accumulation of cultural innovations, such as tools or knowledge, often follows a punctuated pattern [1–4,8,16]. This seems to be particularly true–and puzzling–with regard to the archaeological record of stone tools during the evolution of hominids. Moreover, there appear to be at least two types of punctuated changes apparent in the archaeological record: large and rare punctuation events that encompass major cultural upheavals, and minor, more frequent punctuations, that are smaller in scope and occur between relatively short periods of stasis. Previous accounts have attributed both punctuated patterns to such external factors as environmental or cognitive changes [16,17,22–25], suggesting that these changes affect the parameters that determine the cultural steady state of the hominid population.

In previous work, we suggested an alternative explanation that does not invoke external factors, and suggested that such punctuations may be an intrinsic characteristic of cultural evolution, deriving from the complex cognitive and behavioral mechanisms underlying the innovation process. We suggested that punctuations in the cultural record could represent waiting times between novel innovations that trigger and provide cultural niches for further, related, innovations [33]. This process may underlie the minor, more frequent, cultural changes described above, but is less likely to fully explain the major cultural upheavals occasionally observed in the archaeological record, such as between the Lower, Middle, and Upper Paleolithic. Thus, the question remains: how can we understand the most dramatic shifts in cultural repertoires? Can these major large-scale punctuations be explained as a feature of cultural evolution, as minor, smaller shifts can, or must we invoke external factors to explain them?

Here, we propose a framework in which the largest cultural shifts can result from the process of cultural evolution itself: innovations can themselves alter the underlying rates of cultural gain and loss or change the population size by bringing about changes in food availability, thereby spurring large-scale changes in the cultural steady state. Our model is somewhat unusual in that the parameters that characterize a population are not held constant throughout a simulation. Instead, we suggest a mechanism for these parameters to be changed through the cultural processes that are being modeled. The two drivers of punctuation–technological leaps that facilitate further related innovations, and changes to parameters that alter the cultural steady state, triggering major cultural upheavals–can potentially occur on similar timescales. However, in many cases, such as those demonstrated here, they occur on different timescales, with changes in the cultural steady state occurring rarely, but leading to explosions of cultural change that are typically much larger than changes that are driven by innovations that do not affect the system’s parameters. Notably, one study [56] has previously proposed a model in which a population is drawn towards one of two stable states of cultural complexity and population size, and shifts between the two may occur, driven by the combined dynamics of culture and demography. However, the model in [56] primarily focuses on recurring shifts between two particular states, and also does not account for the two scales of magnitude of punctuated cultural changes that are observed in the archaeological record.

Here we have considered game-changing innovations of two types—those that increase the biological carrying capacity and those that decrease the rate at which tools are lost. To demonstrate the scope of possibilities, loss of a game-changing innovation was implemented only in the latter section of the results, with regard to innovations that change the rate of tool loss. The qualitative result of the loss of an innovation that increased biological carrying capacity is similar: the cultural repertoire shrinks towards its new steady state.

Although loss of both types of game-changing innovations is conceivable, their likelihoods may differ: an innovation that affects the biological carrying capacity is likely to be one that significantly influences the population’s subsistence strategy, and to be known by many individuals. Its loss is thus unlikely, and, moreover, the results of such loss (for example, a smaller food supply) are likely to be evident within a short time period, perhaps less than a generation, while some notion of the innovation still exists in the population, possibly facilitating its re-invention if it is lost. On the other hand, knowledge that reduces the rate of cultural loss, such as writing, may be concentrated in small segments of the population, and thus may be more readily lost. The outcome of such a loss–the subsequent loss of other innovations at a somewhat increased rate–would play out quite slowly, over many generations, and the cost of the loss would be more likely to go unnoticed by the population as a whole than the loss of a subsistence strategy, thus decreasing the likelihood of a response such as re-invention of a similar trait while its notion still exists.

For the human cultural record, it is important to consider punctuation in the context of its magnitude and the timescale on which it occurred, since different timescales can lead to inferences of punctuation that may derive from quite different processes. A note of caution is due in this regard: some interpretations of cultural records as being punctuated may derive from the fact that cultural records are in many cases incomplete. Thus, a process of gradual continuous cultural change may leave an archaeological trace that is sparse, creating a false impression of punctuation. We are agnostic with regard to the interpretation of any particular cultural record, but point out that, depending on parameter values, our model can give rise both to punctuated cultural trajectories and to continuous gradual change as can be seen in panel B of Fig 2 (see also [33]).

Our model demonstrates that interpreting the existence of a large-scale punctuated cultural change as evidence for a biological change in the human population might be unwarranted, given that an exclusively cultural framework such as ours is capable of explaining such changes in the cultural repertoire through realistic cultural modification of the parameters that affect cultural evolution.

As noted above, different processes can underlie a change in steady state that is driven by cultural dynamics. For example, in our model and in others, an increase in population size is often assumed to increase the rate of innovation and to decrease the rate at which culture is lost. Such an increase in rates of innovation may induce further inventions that alter the parameters of cultural evolution. For example, one could imagine a ratchet-like accelerating process in which the changes to the steady state become more frequent as the population grows and culture accumulates. A similar result would be achieved if the likelihood of a steady-state-changing innovation were dependent on cultural complexity in itself. Such a case may be represented by archaeological epochs, whose boundaries are defined by the lithic technologies that were practiced during each of them: these epochs decreased in duration as cultural complexity and population sizes increased, from a very long Lower Paleolithic (~3.3 Mya–300 kya), to a shorter Middle Paleolithic (300–45 kya), to an even shorter Upper Paleolithic (50–10 kya).

Previous models have demonstrated that a process of tool invention via combination of existing tools may lead to accumulation of tools at a polynomial or even exponential rate [9,33,38,51]. The results presented here suggest an alternative mechanism that would produce such dynamics: a rapid, nonlinear increase in the cultural repertoire may be a result of a ‘moving target’ of the cultural steady state and/or related parameters, if these parameters change relatively often before a steady state is achieved. Thus, for example, if cultural innovations bring about changes in population size and occur fairly frequently, and if the rate of cultural accumulation is dependent on population size, the resulting trajectory would increase nonlinearly in time.

Many intuitions about human cultural change and hypotheses that emerge from the archaeological record can be tested using a framework such as the present one. In addition to the advent of agriculture and writing suggested above, our model could accommodate such game-changing innovations as the printing press [57], techniques for an accurately transmitted oral tradition [58], modern medicine [59], and the Green Revolution in agriculture, including synthetic fertilizers and new cereal strains [60].

An aspect of cultural evolution that is beyond the scope of the current study, but whose study in a framework such as ours could prove insightful, is the way in which the cultural repertoire is affected by the functional relationships between novel technologies and existing ones. Thus, for example, some inventions seem to take on the role of earlier technologies and lead to their complete or near-complete replacement, as has occurred with recent changes in technologies of digital communication and data storage. Other technologies, despite providing an alternative to traditional methods, continue to exist side by side with them for millennia, as, for example, in the technologies of production of ceramics, olive oil, cheese, and wool. Future empirical and theoretical research could investigate the persistence of multiple technologies associated with the production of these and similar goods. For example, earlier ceramic production methods might persist because knowledge of or access to the new technology is confined to a subset of the population; alternatively, the earlier technology might persist because it is more efficient for small-scale home production even if the new technology is more effective for larger-scale production. A particularly interesting avenue of exploration along this path could incorporate the functional role of new technology with the historical fate of its bearers: some technologies, particularly subsistence technologies, may be superior to previous ones to an extent that they either replace them directly or replace them via replacement of the groups that fail to adopt the new technology. Some would argue that this was the fate of Neanderthals at the end of the Middle Paleolithic (see, e.g., [61,62]) and of hunter-gatherers in Europe following the spread of agriculture (e.g. [63–65]).

Although our model demonstrates that extrinsic changes, such as environmental and cognitive changes, are not necessary to explain large-scale bursts of cultural accumulation, it is likely that cultural, environmental, and genetic changes all play a role in large-scale changes in human history. Moreover, their dynamics are likely to be intertwined in many cases (e.g., [66]); a promising avenue of future exploration would be the study of dynamics in which biology, environment, cultural norms, and innovative processes co-evolve and feed back on one another.
